# Application of nanotechnology in the diagnosis and treatment of bladder cancer

**DOI:** 10.1186/s12951-021-01104-y

**Published:** 2021-11-27

**Authors:** Yadong Xu, Cheng Luo, Jieqiong Wang, Lingwu Chen, Junxing Chen, Tianfeng Chen, Qinsong Zeng

**Affiliations:** 1 Department of Urology, The First Affiliated Hospital, Sun Yat-Sen University, Guangzhou, 510080 China; 2 Department of Urology, Guangzhou First People’s Hospital, Guangzhou, China; 3 Department of Chemistry, Jinan University, Guangzhou, 510632 China

**Keywords:** Nanotechnology, Bladder cancer, Nanoparticles, Diagnosis, Therapy

## Abstract

Bladder cancer (BC) is a common malignancy in the genitourinary system and the current theranostic approaches are unsatisfactory. Sensitivity and specificity of current diagnosis methods are not ideal and high recurrence and progression rates after initial treatment indicate the urgent need for management improvements in clinic. Nanotechnology has been proposed as an effective method to improve theranosis efficiency for both non-muscle invasive bladder cancer (NMIBC) and muscle invasive bladder cancer (MIBC). For example, gold nanoparticles (AuNPs) have been developed for simple, fast and sensitive urinary sample test for bladder cancer diagnosis. Nanoparticles targeting bladder cancers can facilitate to distinguish the normal and abnormal bladder tissues during cystoscopy and thus help with the complete removal of malignant lesions. Both intravenous and intravesical agents can be modified by nanotechnology for targeted delivery, high anti-tumor efficiency and excellent tolerability, exhibiting encouraging potential in bladder cancer treatment. Photosensitizers and biological agents can also be delivered by nanotechnology, intermediating phototherapy and targeted therapy. The management of bladder cancer remained almost unchanged for decades with unsatisfactory effect. However, it is likely to change with the fast-developed nanotechnology. Herein we summarized the current utility of nanotechnology in bladder cancer diagnosis and treatment, providing insights for the future designing and discovering novel nanoparticles for bladder cancer management.

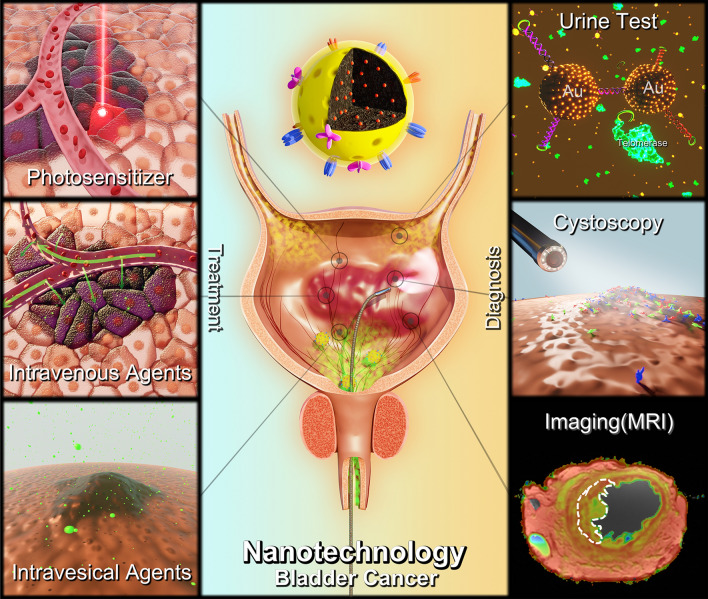

## Background

Bladder cancer (BC) is a common malignancy with high incidence and mortality. It is estimated that there will be 83,730 new cases and 17,200 deaths of bladder cancer in the United States, 2021 [1]. According to the invasive depth, BC is divided into non-muscle invasive bladder cancer (NMIBC) (diagnosed in 75% new cases) and muscle invasive bladder cancer (MIBC) or metastatic cancers (diagnosed in 25% new cases) [2]. Cystoscopy and urine cytology are the gold standard for BC diagnosis; Magnetic resonance imaging (MRI) has been suggested for imaging examination of the staging. Transurethral resection of bladder tumor (TURBT) is the primary method for the initial treatment of NMIBC, and intravesical instillations of chemotherapy or immunotherapy after TURBT are aimed for disease recurrence and progression prevention. MIBC is routinely treated by radical surgery and urinary diversion with or without adjuvant therapies. However, radical cystectomy brings significant advert effects and impairs the quality of life. Thus, bladder-sparing treatment with the combination of TURBT, radiation and chemotherapy are also commonly administrated in clinical practice [3, 4]. With the development in understanding of bladder cancer biology, targeted treatments and immunotherapies have shown significant potential for BC treatment [5]. Erdafitinib, a kind of fibroblast growth factor receptor (FGFR) inhibitor, and Avelumab, an anti-PDL1 agent were approved by the FDA for advanced MIBC [6, 7]. Several other targeted or immunotherapy agents are in clinical trials [8].

Despite all these theranostic strategies, the treatment and diagnosis of BC are unsatisfactory. Urine cytology, MRI for detecting or staging of BC are of limited sensitivity; cystoscopy is invasive, costly and uncomfortable [9–11]. In spite of multiple efforts to constrain the recurrence and progression, the reported recurrence rate was 50%-90% within five years for NMIBC[12, 13]. Therefore, other techniques are urgently needed to improve the diagnostic and therapeutic efficiencies in BC treatments.

**Fig. 1:**
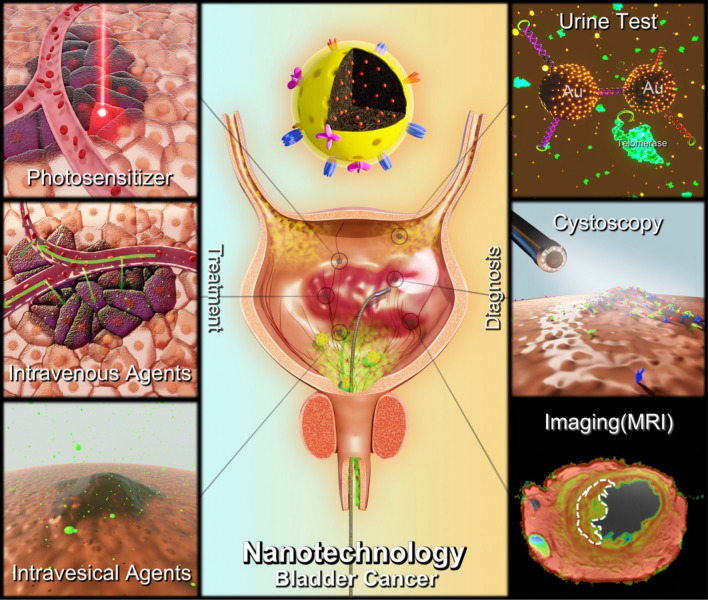
The application of nanoparticles in bladder cancer diagnosis and treatment

Nanotechnology has been proposed as a novel technique in the management of BC and reported in lots of studies (Table 1). Targeting at the shortcomings of current theranostic approaches, nanotechnology shows great potential for clinical utilization. NP carriers can improve the aqueous solubility, stability, bioavailability or other properties as needed for primary agents; and surface modification with tumor cell targeted peptide or antibody can endow the system with recognition ability for tumor cells [14]. Well-designed NPs can detect cancer biomarkers with satisfactory accuracy and specificity or improve the efficiency of current theranostic modalities. NP based delivery systems can also increase active cellular uptake and targeted localization to tumor sites, which decreases the damage to normal cells and reduces side effects. In addition, NPs are utilized to prolong the residence time of intravesical agents for better antitumor efficiency [15, 16]. Moreover, nanotechnology can integrated with other novel technologies (e.g. phototherapy, radiotherapy) to further improve effectivity. With the further understanding and development of nanotechnology and ontogenetic mechanisms, more efficient and reliable systems for the diagnosis and treatment of BC are expected to arise. Tremendous successes have been made in preclinical studies for bladder cancer in recent years. Thus, we feel that the field has progressed to a point where a review article like ours will be of general interest and appeal to a wide readership. Several groups also reviewed the application of nanotechnology in urological or bladder cancers during the past three years [17–19]. However, most of their studies were summarized based on the basic structure of nanoparticles, and our review focused on the different diagnosis and treatment approaches and the potential utilities of nanotechnology in these processes (Fig. 1), which may be helpful for future designing and exploring novel nanosystems for BC management. A large proportion of different studies were mentioned between our review articles and others, so we believe all these review articles are informative and can provide different perspectives for the readers.

**Table 1:** The current application of nanotechnology in bladder cancer diagnosis and treatment

	Categories	Examples	Applications	Refs.
Diagnosis	Urine sample	AuNPs	Detect hyaluronidases	[21]
		Gelatin-Modified AuNPs	Detect gelatinases	[24]
		AuNPs	Detect unamplified HURP RNA	[25]
		AuNP-H1 and AuNP-H2 probes	Detect telomerase activity	[28]
	Cystoscopy	Surface-Enhanced Raman Scattering (SERS) Nanoparticles	Raman Cystoscopy	[40]
		PLZ4-Nanoporphyrin	Fluorescent signal	[41]
	Imaging	Ferromagnetic nanocubes Loaded Glycol Chitosan	MRI contrast agents	[45]
		Cyc6-Gd2O3-FITC-MSN	MRI contrast agents and fluorescent signal	[46]
		Ferumoxtran-10	MRI contrast agents for nodal staging	[47]
Treatment	Intravesical instillation	BCG -CWS NPs	Intravesical instillation	[60]
		BCG Loaded Chitosan NPs	Intravesical instillation	[61]
		PLZ4@SeD	Intravesical instillation	[64]
		Epirubicin Loaded Polymeric NPs	Intravesical instillation	[67]
		Cisplatin Loaded Poly (L-Aspartic Acid Sodium)	Intravesical instillation	[71]
		Pt − Fe − PNs	Intravesical instillation	[72]
		Deguelin Loaded DMP	Intravesical instillation	[73]
		Multiple Cationic NPs Encapsulating Mitomycin C	Intravesical instillation	[74]
		Doxorubicin Loaded Cationic NPs	Intravesical instillation	[68]
		NP-Bel-PGON	Intravesical instillation	[75]
		PTX-Loaded Gelatin NPs	Intravesical instillation	[77]
		PTX&DTX Loaded Polymeric Micelles	Intravesical instillation	[78]
		PTX-Loaded dHPGs Micelles	Intravesical instillation	[79]
		nab-PTX	Intravesical instillation	[80]
	Intravenous agents	nab-PTX	Intravenous administration	[88]
		DiD And Chemotherapeutics Loaded Targeting PLZ4 Micelles	Intravenous administration	[89]
		PTX Loaded Targeting Micelles	Intravenous Administration	[90]
	Photosensitizers	Mesoporous Silica-Coated Nayf4 NPs	Photodynamic therapy	[97]
		5-Aminoevulinic Acid Loaded Copolymers	Photodynamic therapy	[98]
		Black Titanium Dioxide NPs	Photodynamic therapy	[99]
		FGF1-Coated AuNPs	Infrared-induced thermal ablation	[100]
		BPN-BBTD NPs	Fluorescence imaging and photothermal therapy	[101]
	Photosensitizers	HSA-MnO_2_-Ce6 NPs	O_2_-generating and photodynamic therapy	[102]
	Biological agents	Tumor-Targeting Moiety Decorated Cationic Liposome Encapsulating RB94 Plasmid	Tumor suppress gene and synergism with chemotherapy	[104]
		dsP21-322–20’ F Loaded Lipid NPs	Intravesical instillition for P21 induction	[106]
		Surviving siRNA Loaded Chitosan Modified PLGA NPs	Intravesical instillition to inhibit surviving expression	[105]
Theranosis	NA	Fe3O4@PDA-VCR-FA SPs	MRI contrast agent and photothermal therapy	[109]
		Nanoclusters of UCNP and AuNR	Fluorescent signal and optoporation-assisted chemotherapy	[110]

*AuNPs* Gold nanoparticles, *HURP RNA* hepatoma upregulated RNA, *MRI* magnetic resonance imaging, *MSNs* mesoporous silica nanoparticles, *BCG* Bacillus Calmette-Guerin, *CWS *cell wall skeleton, *PNs* polymeric NPs, *DMP* DOTAP and monomethoxy poly(ethylene glycol)-poly(ε-caprolactone) hybrid nanoparticles, *PTX* Paclitaxel, *DTX* doxetaxel, *UCNP* upconversion nanoparticle, *AuNR* gold nanorod, *NA* not applicable

## Bladder cancer diagnosis and nanotechnology

### Urine sample

Non-invasive urine cytology for NMIBC is an important diagnostic technique and widely used in clinical procedures. By analyzing cells from the urine sample, it is much easier and more comfortable than tissue biopsy. It is also a gold standard for bladder cancer diagnosis attribute to its high specificity. However, poor sensitivity capped its application in clinic [20]. Further improvement in its diagnostic sensitivity is needed. The urine samples from BC patients may contain different biomarkers from normal ones and these biomarkers may be detected by nanotechnology. These nanoparticles may have better sensitivity and specificity than urine cytology.

**Fig. 2:**
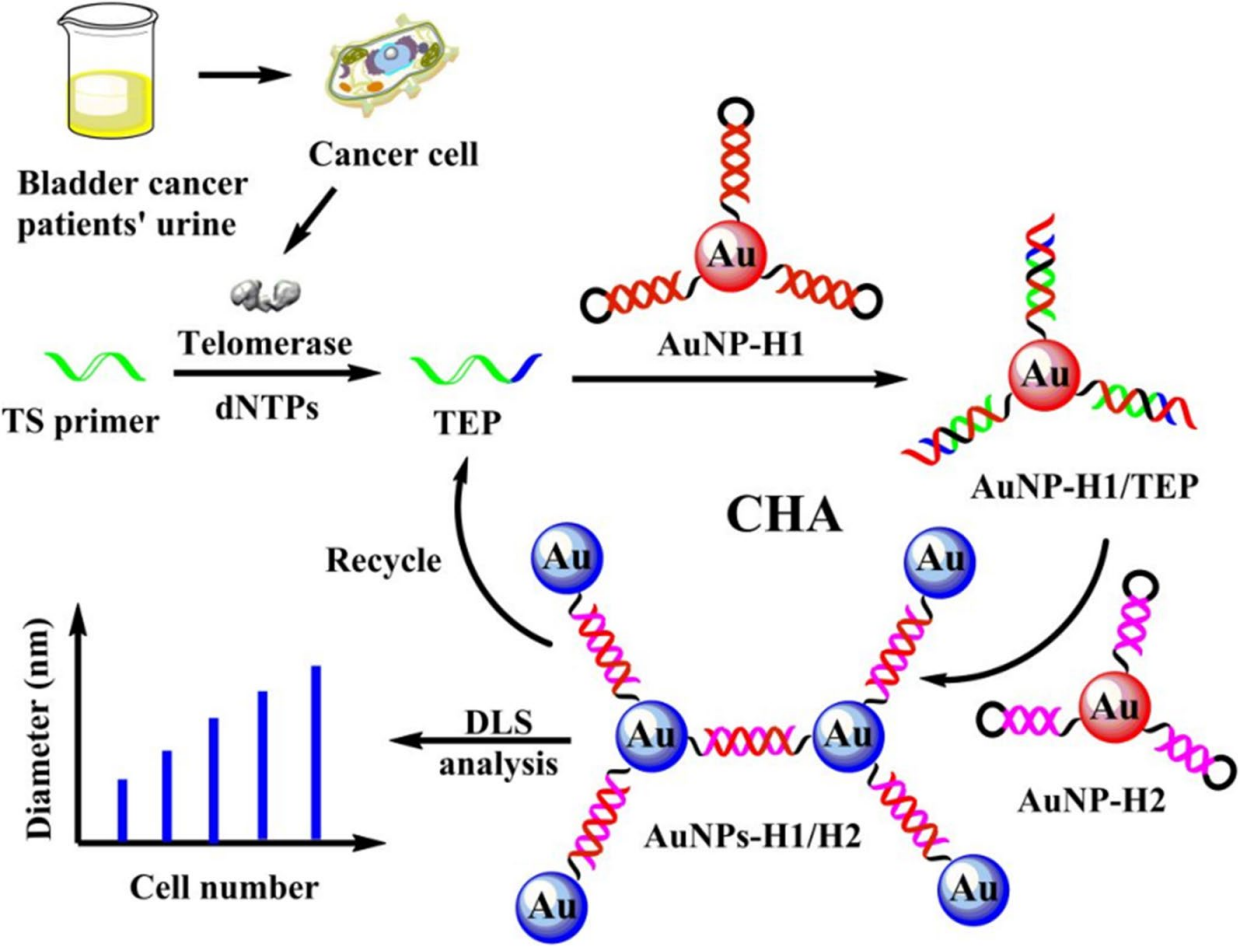
Schematic diagram of AuNPs based assay for telomerase activity detection [28] ( Copyright 2020, reproduced with permission from American Chemical Society)

Hyaluronidases are extracellular matrix-digesting endoglycosidases and they are elevated in the urine of bladder cancer patients [21]. Gold nanoparticles (AuNPs) are characterized by strong surface plasmon resonance (SPR) due to their size and interparticle distances. From dispersion to aggregation, AuNPs would couple with a visible color shift from red to blue [22, 23]. Nossier et al. applied this exclusive property of AuNPs to directly detect hyaluronidases in the urine of bladder cancer patients. With cetyl trimethyl ammonium bromide (CTAB) stabilized AuNPs and hyaluronic acid (HA) added to the urine samples, the present of hyaluronidases was reflected by the unchanged red color of AuNPs[21]. The AuNP based method showed better sensitivity (82.5 vs. 65%) and comparable specificity (96.1 vs. 96.1%) compared with traditional zymography method [21]. Other colorimetric gelatin-modified AuNPs were proposed to quantify the total concentration of gelatinases, which serve as another urinary marker of BC. Gelatinases digest the gelatin capping, then gelatin-modified AuNPs will shift from red to blue upon induced by an aggregation inducer. The total gelatinase concentration is quantified by the A625/A530 absorption ratios [24]. Eissa et al. found the hepatoma upregulated RNA (HURP) in urine could serve as a BC marker with 78.8% sensitivity and 94% specificity by semi quantitative RT-PCR. Then they developed an AuNP assay to directly detect the unamplified HURP RNA for BC diagnosis. The sensitivity and specificity of this assay were 88.5 and 94.0%, respectively [25]. Telomerase is a promising biomarker for tumorigenesis as it is highly expressed in over 85% malignant cancer cells and repressed in most normal somatic cells [26, 27]. Zou et al. developed AuNPs-based systems to detect the tolomerase activity. Two telomerase extension product (TEP) complementary oligonucleotide hairpins were attached to AuNPs to form AuNP-H1 and AuNP-H2 probes. Telomerase can induce the elongation of telomerase substrate (TS) primer, producing TEP, which can further catalyze the forming of stable Au NPs-H1/H2 nanostructures. The activity of telomerase could be reflected by the diameter of AuNP probes detected by dynamic light scattering (DLS) (Fig. 2) [28]. These AuNPs based assay can significantly improve the diagnostic sensitivity of urine sample, especially in low-grade and superficial BCs. However, none of these AuNPs entered clinical use at the present. Firstly, the use of urine sample for BC diagnosis is not fully elaborated yet, so the biomarkers in urine detected by AuNPs may bring some concerns for clinicians. This situation is about to change as a large amount of related studies emerging in recent years [29]. Secondly, almost all AuNPs based assays need sample preparation for detection, and some of these prepare and detect processes may be complicated. Thirdly, the large scale synthesis of AuNPs for clinical use may be difficult and the developed assay might be costly. All these reasons may hinder the application of AuNPs in clinic. Further exploration and efforts are needed for successful translation of AuNPs.

### Cystoscopy

**Fig. 3:**
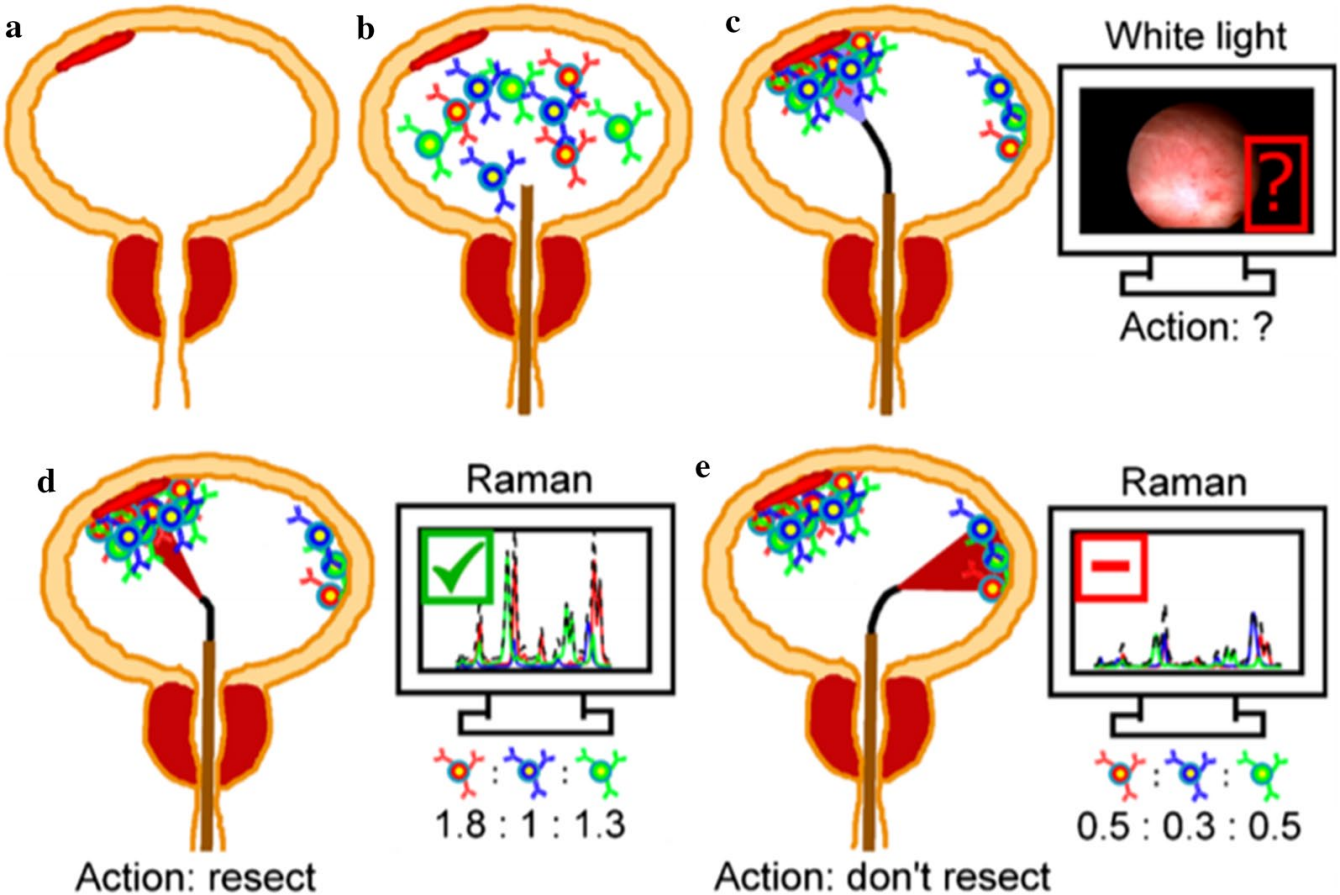
Schematic diagram of SERS NPs application in clinic. **a** Red tissue represents potential NMIBC. **b** SERS NPs is administered before cystoscopy. **c** Flat NMIBC is difficult to identify with WLC, resulting in possible incomplete resection of tumor lesions. **d**, **e** Raman endoscopy guided transurethral resection [40] ( Copyright 2018, reproduced with permission from American Chemical Society)

Right now, white light cystoscopy (WLC) is the most common method for BC diagnosis and surveillance. A flexible camera was inserted into bladder through urethra and image inside bladder could be observed by the urologist. When a suspicious lesion was identified, a biopsy or transurethral resection (TUR) could be done to further assess or treat the neoplasm. Other than discomfort and risk of infection for patients, there are some other drawbacks for WLC. Detecting carcinoma in situ (CIS) would be a great challenge or even impossible, as well as for some small or satellite tumors [30–32]. Incomplete resection of NMIBC and the following high recurrence rate are the common consequences for WLC [33]. A lot of cystoscopic techniques have been implied to ameliorate the visualizing of tumors such as narrow band imaging (NBI) and blue light cystoscopy (BLC). BLC utilizes photoactive agents like 5-aminolaevulinic acid (5-ALA) and hexaminolevulinate (HAL) to instill into the bladder before the endoscopy procedure [34, 35]. Tumor cells are prone to absorb more agents and emit red fluorescence when stimulated by blue light [36]. NBI is a kind of optical image enhancement technology that filters white light into blue (415 nm) and green (540 nm) spectrum. Since the strong absorbance of hemoglobin, NBI creates great contrast between blood vessels and other tissues. Bladder tumors are pinpointed by the vascular abundant character by NBI detection [37]. These techniques are showed to have better sensitivity than WBL, but the specificity may be poor [38, 39]. Nanotechnology can also facilitate the diagnosis performance of cystoscopy. Davis et al. developed intravesical surface-enhanced Raman scattering (SERS) NPs which targeted to bladder cancer to help differentiate bladder tissue as benign or malignant with the endoscope system (Fig. 3) [40]. Nanotechnology may also improve the specificity of photoactive agents to tumor cells and thus provide increased specificity for carcinoma detection. PLZ4, identified by Zhang et al. as a BC cell specific peptide, was applied to form PLZ4-nanoporphyrin. It was proven to be specifically restricted to bladder cancer cells, as the fluorescence signal of cancer cells was much higher than that of normal cells [41].

### Imaging

Ultrasound, computed tomography (CT) and MRI are the most common imaging techniques in clinic. They are useful in the carcinoma diagnosis and staging. As for BC, ultrasound is a noninvasive and cost-effective method for initial diagnosis. CT and MRI are mainly used to evaluate the tumor staging, including local tumor invasion (T stage), lymph nodes involvement (N stage) and metastases state (M stage). But the staging accuracy for the primary tumor is not reliable, especially after TURBT [42–44]. Accurate staging is crucial for the formulation of treatment strategies and prognosis for BC patients. A better staging method is required for the clinical practice. Contrast agents are a useful method to improve the performance of imaging techniques. Delicately manufactured nanoparticles could serve as contrast agents and resulted in high resolution pictures. Ferrimagnetic iron oxide NPs are not widely utilized as MRI agents due to the magnetic memory under an external magnetic field despite their properties of hyperthermia and high contrast. Thus, Key et al. manufactured multimodal NPs by loading 22 nm ferromagnetic nanocubes (NCs) into BC specific peptides modified glycol chitosan nanoparticles (pMCNP). Reduced nonspecific binding, excellent accumulation in tumors and long retention time make them great MRI contrast agents [45]. Sweeney et al. reported the application of mesoporous silica nanoparticles (MSNs) which functionalized with a BC specific peptide, cyc6, and incorporated by Gd_2_O_3_ as MRI contrast agents. Due to the effective binding to the tumor cells of modified MSN, the tumor boundaries were more clear in the T1- and T2-weighted MRI as well as in visualization by fluorescent cystoscopy [46].

**Fig. 4:**
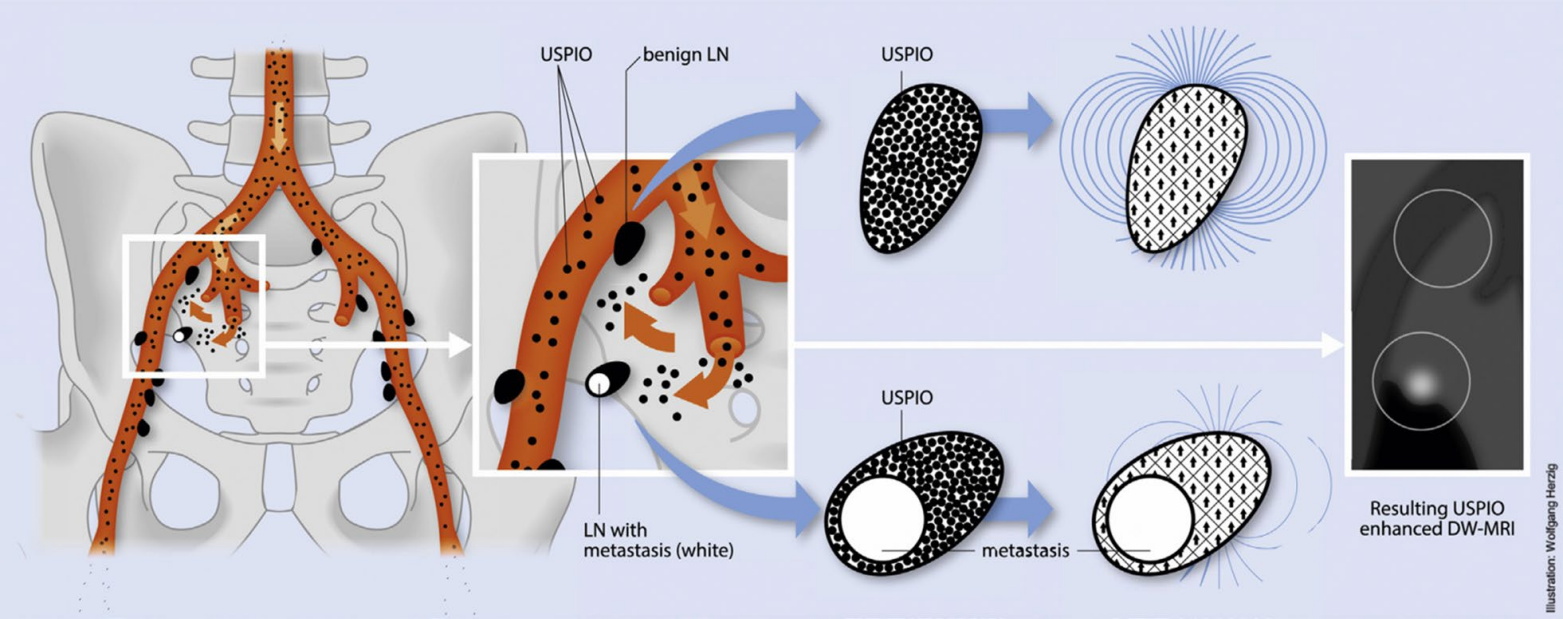
Schematic diagram of USPIOs assisted MRI imaging for nodal staging [48] ( Copyright 2013, reproduced with permission from Elsevier)

Other than local tumor detection, nanotechnology is also used in lymph node evaluation. Currently, for advanced or metastatic BC, the assessment of nodal staging depends on the size and contrast enhancement of lymph nodes displayed in imaging modalities such as MRI or CT. However, an overlap in size was discovered in benign and malignant nodes. Besides, metastatic and normal lymph nodes without an enlargement in size might share similar contrast enhancement [44]. Therefore, other techniques are necessary for improved differentiation between normal nodes and metastasis. Ultra-small superparamagnetic iron oxide (USPIO) particles, used as a contrast material for MRI, were reported to examine the properties of normal-sized pelvic lymph nodes [47]. The quantity of macrophages in lymph nodes determined the signal density on T2 and T2*-weighted MRI, with USPIO injected intravenously. Macrophages in normal nodes could swallow USPIO particles, which resulted in the signal decrease of MRI. However, the signal drop was not present in metastatic lymph nodes due to the absence of macrophages. The diagnostic ability of USPIOs-enhanced MRI was examined in nodal staging of BC. The sensitivity and specificity developed from 92 and 76% with conventional evaluation criteria to 95 and 96% with the novel MRI technique. Metastases were discovered even in normal-sized lymph nodes (< 10 mm) with node–node comparison with histologic findings [47]. Another study focused on pelvic lymph nodes of normal size with genitourinary malignancies. In the 2993 resected lymph nodes, 54 metastases were demonstrated, even in nodes with a short axis smaller than 5 mm. So, USPIOs-enhanced MRI can significantly improve the diagnostic accuracy in genitourinary cancers with a N0 stage (Fig. 4) [48]. However, this technique requires thirty minutes of administration and expertise for specialized interpretation, which restricts its use in clinical practice [49]. Therefore, modified NPs with advanced functions are in request for convenient use and accurate prediction in the nodal evaluation of BC.

Positron Emission Tomography (PET) is another widely used noninvasive diagnostic imaging technique in clinic. Based on molecular biology, ^18^F-fluorodeoxyglucose (FDG) PET combined with CT (^18^F-FDG PET/CT) enables us to evaluate the metabolic activity of cells, which could serve as significant distinction between normal and malignant cells [50]. ^18^F-FDG PET/CT has been widely used for tumor detection and staging, such as lung, esophageal, cervical, lymphoma and breast cancers [51]. However, due to the urinary excretion of FDG, it strongly interferes with the primary tumor detection of BC. So PET/CT is rarely used in primary bladder tumor evaluation. It is useful for accurate detection of metastases in BC.

To overcome the interfere of urinary excretion of FDG, ^11^C-choline, ^11^C-methionine and ^11^C-acetate were studied as alternative tracer for primary BC detection in PET/CT, [52, 53]. In addition, a lot of nanoparticles are also constructed as potential radiotracers for PET/CT [54–56]. Right now, few studies reported the utilization of nanoparticles in BC for PET/CT detection, but we believe they may help overcome the obstacles of current tracers and worth further exploration.

## Bladder cancer treatment and nanotechnology

### Delivery of intravesical drugs

Intravesical instillation therapy for BC has a number of advantages including high local drug concentration and minimal systemic toxicity given to the localized exposure to the drugs. Bacillus Calmette-Guerin (BCG) and chemotherapy agents are the most common used drugs. However, local and systemic side effects, urinary dilution, unsatisfied penetration to the bladder wall and instability of agents in low urinary PH greatly limited the drug efficiency [57]. Nanotechnology could serve as a solution for these obstacles.

#### BCG loaded nanoparticles

Live BCG instillation intravesically is a postoperatively adjuvant treatment for NMIBC, especially for high-grade tumors and carcinoma in situ. However, the side effects, such as significant urinary symptoms, systemic infections, and sepsis, are still a danger leading to the suspension of BCG therapy [58]. The BCG cell wall skeleton (BCG-CWS) is the active immunoadjuvants component of BCG and can be used to replace live BCG [59]. However, the application of BCG-CWS in clinic is restricted due to the low uptake by cancer cells and poor solubility of BCG-CWS. To overcome this obstacle, nanoparticles loaded with BCG-CWS (CWS-NP) were produced by the liposome evaporated via the emulsified lipid (LEEL) method. CWS-NP/LEEL system was demonstrated to exert antitumor effect without observable side effects in rat model cells and human cells. In addition, the CWS-NP/LEEL could enhance Th1 immunity and reduce Th2 cells in the Th1/Th2 balance[60]. Cationic chitosan NPs as a delivery system for BCG were also evaluated. Due to the positive surface charge, high tumor targeting, and nano range size, BCG loaded cationic NPs were presented with an improved delivery rate by overcoming the bladder permeability barrier and possessing high antitumor efficiencies [61].

#### SPIONs and SeD loaded nanoparticles

**Fig. 5:**
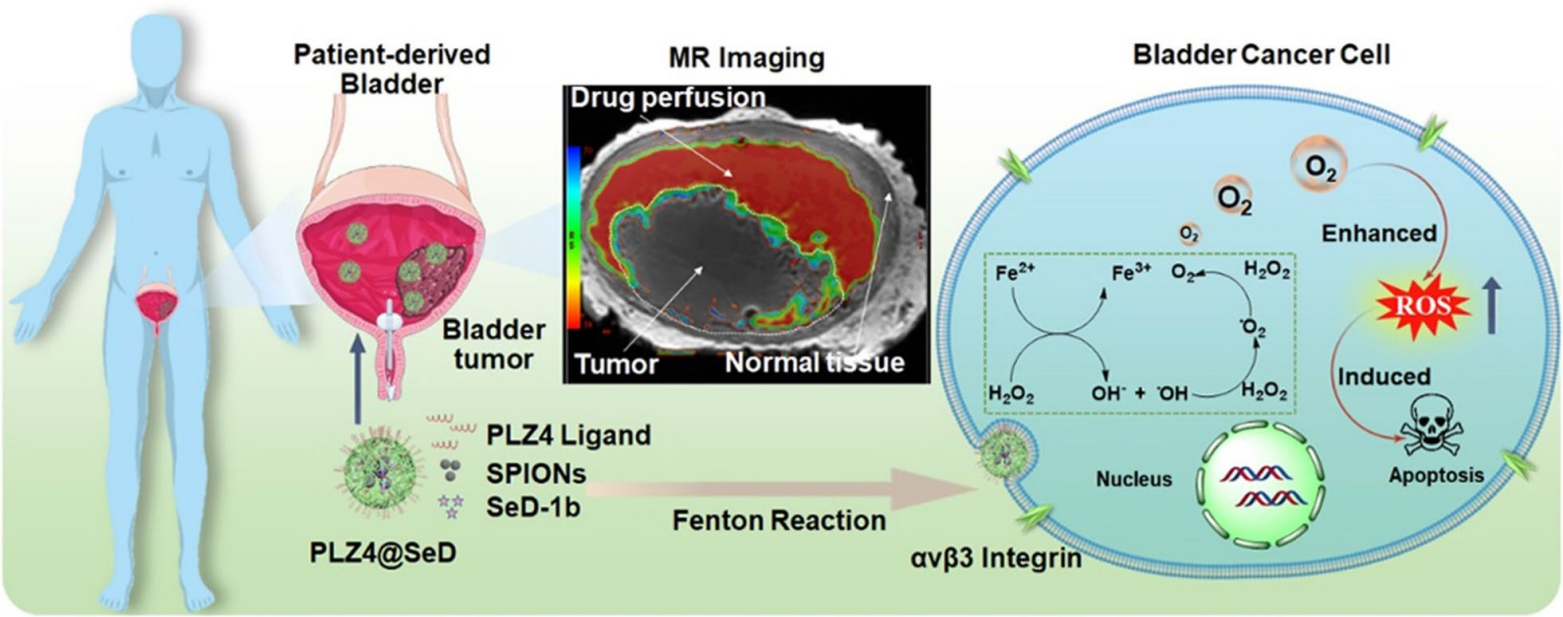
Schematic diagram of PLZ4@SeD NPs design and MRI-guided chemotherapy through ameliorating hypoxia [64] (Copyright 2021, reproduced with permission from Elsevier)

Local hypoxia has been demonstrated as an essential characteristic of tumor microenvironment and contributed significantly to treatment resistance [62, 63]. Weiqiang et al. developed superparamagnetic iron oxide nanoparticles (SPIONs) loaded nanoscale oxygen generator (PLZ4@SeD) with bladder cancer targeting moiety to release the hypoxia in bladder cancer by intravesical instillation [64]. The nanoparticles showed great targeting ability and tumor permeability to 3 patient-derived bladder tumors and thus enhance the MRI contrast efficiency. They can also generate oxygen through Fenton reaction to release the hypoxic condition, facilitating chemotherapy efficacy (Fig. 5). PLZ4@SeD showed great potential in BC theranosis.

#### Epirubicin and doxorubicin loaded nanoparticles

Epirubicin and doxorubicin are commonly used as adjuvant chemotherapy for intravesical administration. However, the low permeability of intravesical drugs into the bladder wall is an obstacle for the ideal therapeutic effect [65]. Polymeric NPs are able to improve the permeability of intravesical drug and extend the dwell time due to their water-dispersible and mucoadhesive properties [66]. Another study load epirubicin with poly(ethyl-2-cyanoacrylate)(PECA) NPs and demonstrated that this system could provide sustained drug tissue concentrations and significant cytotoxicity in human BC cells [67]. Jin et al. encapsulated doxorubicin (Dox) with another cationic nanoparticle and significantly improved the efficiency of Dox in orthotopic BC model [68].

#### Cisplatin loaded nanparticles

For locally advanced and metastatic BC patients, cisplatin-based chemotherapy may have superior efficacy among other chemotherapeutic agents [69]. However, repeated intravesical instillation with cisplatin may cause chemical cystitis, anaphylactic reactions and other side effects. A prior clinical trial for the usage of cisplatin in NMIBC was stopped for these side effects [70]. Some groups have developed cisplatin-based NPs for potential clinical utilization. Cisplatin NPs were manufactured by utilizing poly(L-aspartic acid sodium) to load cisplatin and were proven to significantly elevate the bladder tissue drug concentration and improve the anticancer activity while reducing the local and systemic side effects [71]. In another group, superparamagnetic iron oxide NPs (SPIONs) and polymeric NPs were combined as versatile carriers to deliver cisplatin (Pt − Fe − PNs). The NPs could initially release cisplatin rapidly and be sustained for over four days in artificial urine in human body temperature. The cytotoxicity of the NPs was indicated in UMUC3 BC cells with IC50 of 32.3 μM. As the SPIONs endow magnetic properties, this system could serve as MRI contrast agents to assist bladder cancer diagnosis. Furthermore, they could also enhance the treatment efficiency by localized hyperthermia induced through external magnetic field [72].

#### Deguelin loaded nanoparticles

**Fig. 6:**
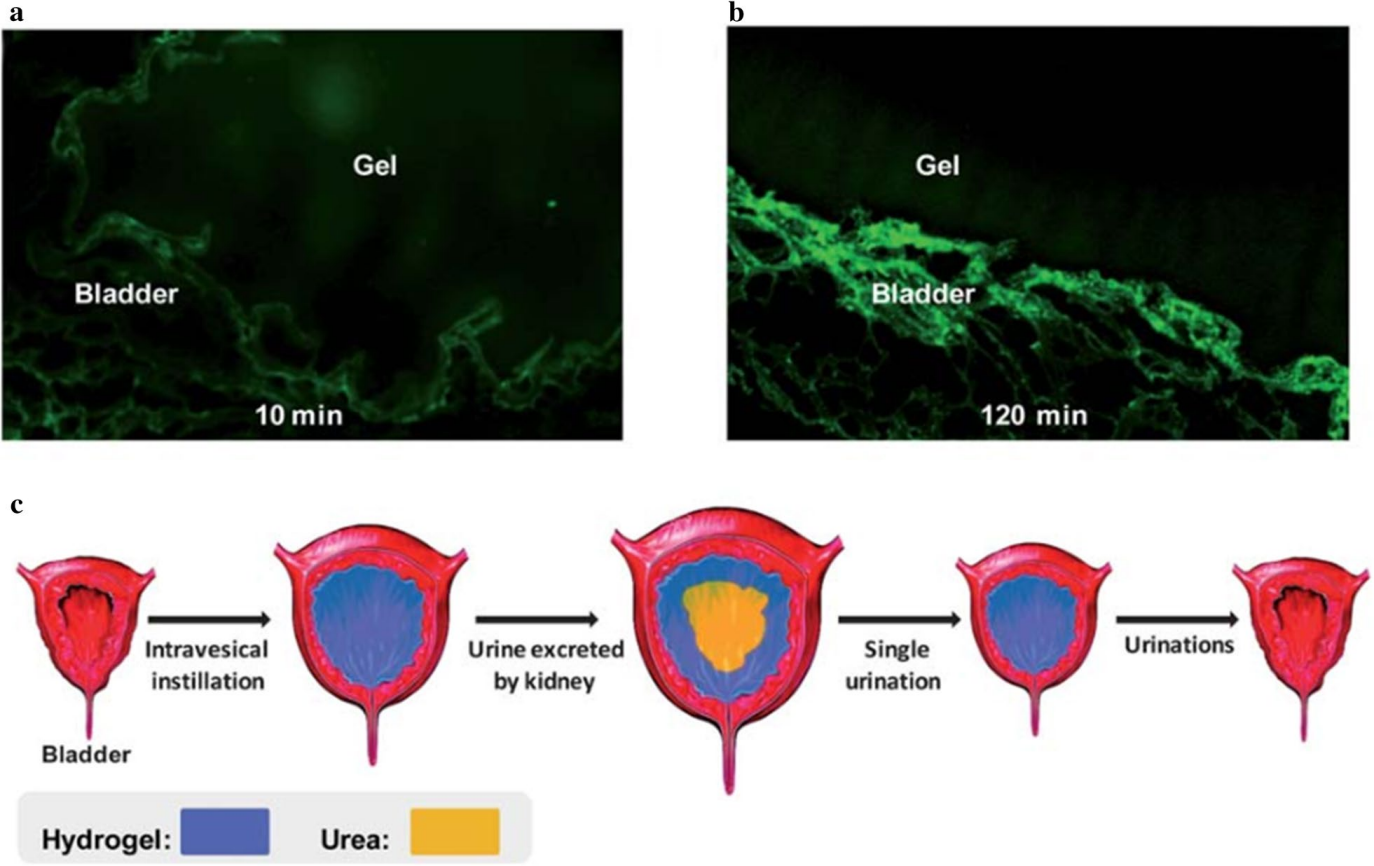
**a** DMP-F gel was intravesically administrated in 10 min. **b** After 2 h, some DMP-F gel dissolved in the urine and some remained pasted on the bladder wall. **c** Schematic diagram of DMP-F gel elimination in bladder during urination [73] ( Copyright 2012, reproduced with permission from Royal Society of Chemistry)

Cationic DOTAP and monomethoxy poly(ethylene glycol)-poly(ε-caprolactone) hybrid nanoparticles (DMP) were developed to deliver deguelin (D/DMP). Deguelin is a potential antitumor drug, and D/DMP improves its water solubility and reduces its neurotoxicity [73]. D/DMP nanoparticles were further incorporated into a kind of thermo-sensitive hydrogel, which endowed its hydrophobic property in body temperature. The developed system significantly increases the dwell time and concentration of deguelin within the bladder (Fig. 6) [73].

#### Mitomycin C loaded nanoparticles

For a better penetration of the bladder wall and uptake by tumor cells, multiple cationic NPs were used to encapsulate Mitomycin C (MMC) including chitosan (CS), CS coated poly-ε-caprolactone (CS-PCL) and PCL coated with poly-ι-lysine (PLL-PCL). These nanoconjugates were documented with favorable antitumor effects [74].

#### Belinostat loaded nanoparticles

**Fig. 7:**
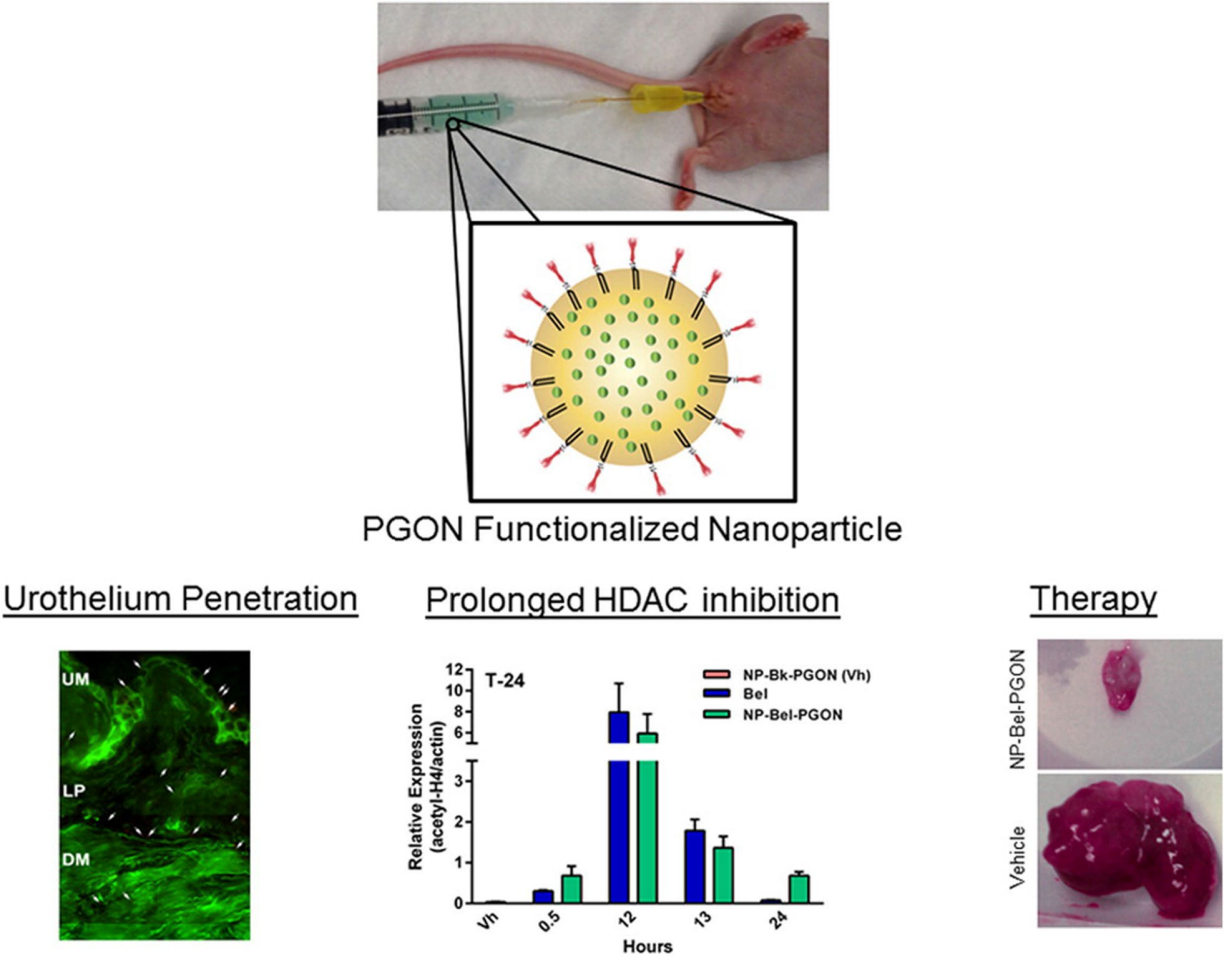
Schematic diagram of PGON functionalized belinostat loaded nanoparticles utilization in bladder cancer treatment [75] ( Copyright 2013, reproduced with permission from Elsevier)

Martin et al. loaded belinostat, a kind of histone deacetylase inhibitor, with poly(lactic-co-glycolic acid) (PLGA) and surface modified with poly(guanidinium oxanorbornene) (PGON), a novel cell penetrating polymer (NP-Bel-PGON). NP-Bel-PGON exhibited better antitumor performance compared with unencapsulated belinostat both in vitro and in vivo (Fig. 7) [75].

#### Paclitaxel loaded nanoparticles

Paclitaxel (PTX) is a kind of antimicrotubule compounds which has great potential for intravesical therapy with its lipophilicity property. However, cremophor EL/ethanol is the commercial formulated solution for PTX, which is proved to reduce the ability of PTX to penetrate into the bladder wall by Knemeyer et al. [76]. To overcome this problem, gelatin, which is characterized by its biocompatibility and hydrophilicity, was utilized to develop a novel formulation to readily release PTX. The PTX-loaded gelatin NPs presented a rapid and almost complete release of the drug and a higher concentration in bladder tissue than in a commercial solvent. Subsequently, the NPs were demonstrated with significant antitumor activity and recommended as a potentially useful carrier for intravesical PTX therapy [77]. Another group encapsulated PTX and doxetaxel (DTX) with polymeric micelles and efficiently enhanced the penetration of PTX and DTX into pig bladder tissue [78]. Via improving the tissue permeability and prolonging the residence time of PTX, hydrophobically derivatized hyperbranched polyglycerols (dHPGs) were used to incorporate PTX for NMIBC intravesical treatment. PTX-loaded dHPGs micelles showed stability in acidic urine and an initial rapid release of the drug. Mucoadhesive dHPGs incorporated PTX showed potent cytotoxicity and significant suppression of tumor proliferation both in vitro and in vivo [79]. Given to the great potential of nanotechnology-modified PTX in the intravesical treatment for NMIBC, nanoparticle albumin-bound (nab)-paclitaxel was evaluated in clinical trials. Phase I trial included 18 BCG refractory NMIBC patients, and showed dysuria as the most common (56%) local toxicity, no grade 2, 3 or 4 adverse events were observed [80]. A further phase II trial reported a 35.7% response rate in 28 enrolled patients [81]. Long-term survival outcomes revealed 61% cystectomy-free survival and 9% cancer-specific mortality, suggesting it as an effective treatment method in high-risk NMIBC patients [82].

### Delivery of intravenous chemotherapeutic agents

For MIBC patients, the treatments include neoadjuvant therapy and the following radical cystectomy [83, 84]. Neoadjuvant chemotherapy prior to surgery or radiotherapy significantly improves the overall survival [85, 86]. For BC patients with metastatic state, the current treatment of cisplatin-based cytotoxic chemotherapy yield disappointed outcome with about only 13–15 months median survival time [69, 87]. Despite the initially good reaction to chemotherapy, some patients eventually relapse and develop with resistant disease. Severe advert events of these chemotherapeutics are also inevitable in clinical practice. New strategies with better tolerance and performance are in needed and being explored. Nanotechnology could serve as a great platform for the modification of these regimens.

Nanoparticle albumin-bound paclitaxel (nab-paclitaxel) as second-line therapy for patients with platinum-refractory BC had got some encouraging results. In a phase 2 clinical trial, forty-eight patients were intravenously injected with nab-paclitaxel 260 mg/m^2^ every 3 weeks, and 27.7% patients had complete or partial response with acceptable side effects [88]. One of the mechanisms explaining the effectiveness of nab-paclitaxel was the high affinity to the tumor-derived protein named SPARC, which offers help to the drug in the binding to the receptor and entry to the tumor interstitium [88]. PLZ4 can specifically bind to BC cells. By applying the property of this peptide in chemotherapy for BC, a multifunctional micelle nanocarrier was constructed by loading imaging agent DiD and therapeutic drugs (paclitaxel or daunorubicin). PLZ4 dramatically improved the uptake of micelles by cancer cells and the enhancement effect was validated in the orthotopic MIBC mice model [89]. In clinic, the application dosage of paclitaxel with Cremophor EL/alcohol for intravenous chemotherapy was 175 mg/m^2^. Micelles with PLZ4 increased the dose of paclitaxel to 260 mg/m^2^ by decreasing toxicity and targeting delivery, which could improve cancer control and overall survival [90].

### Delivery of photosensitizer

**Fig. 8:**
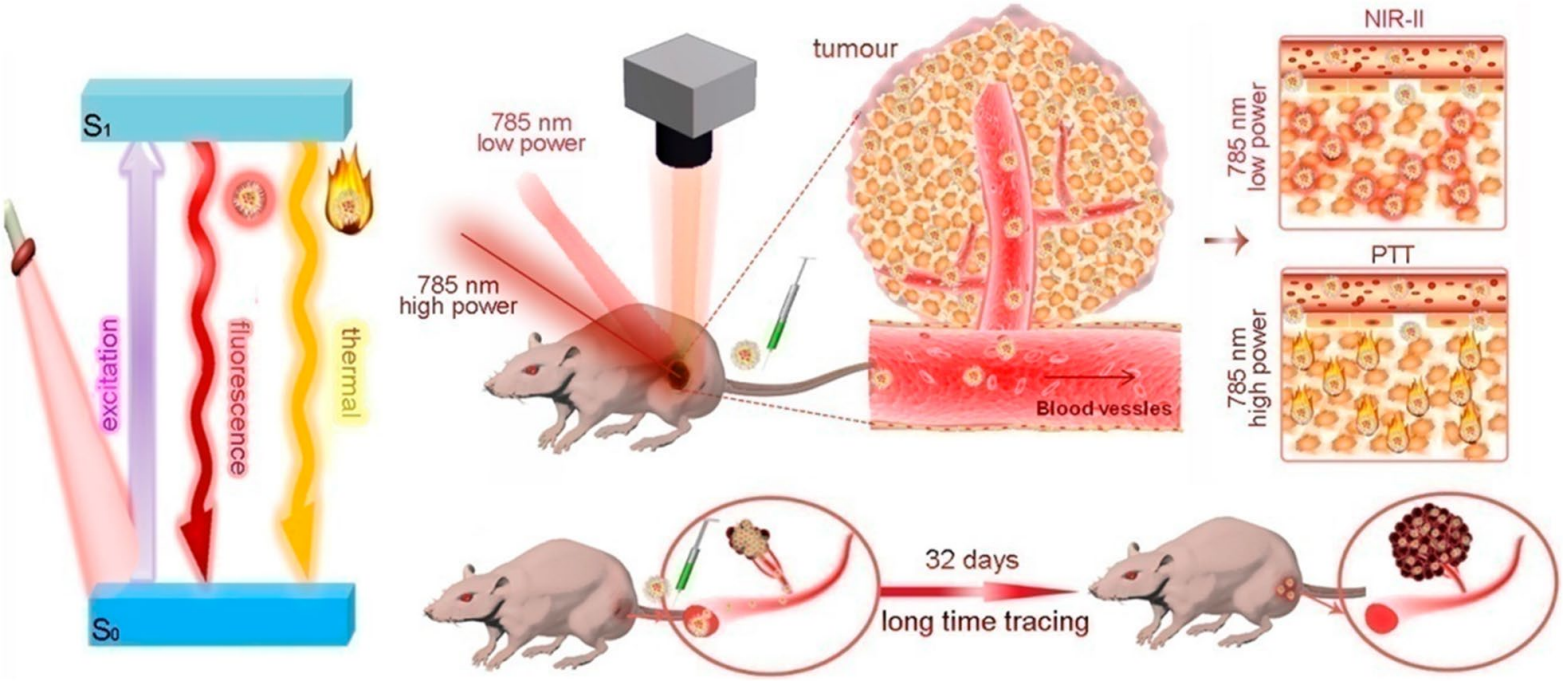
Schematic diagram of BPN-BBTD NPs for in vivo fluorescence imaging, photothermal therapy and long-term tracing of tumors [101] ( Copyright 2018, reproduced with permission from American Chemical Society)

**Fig. 9:**
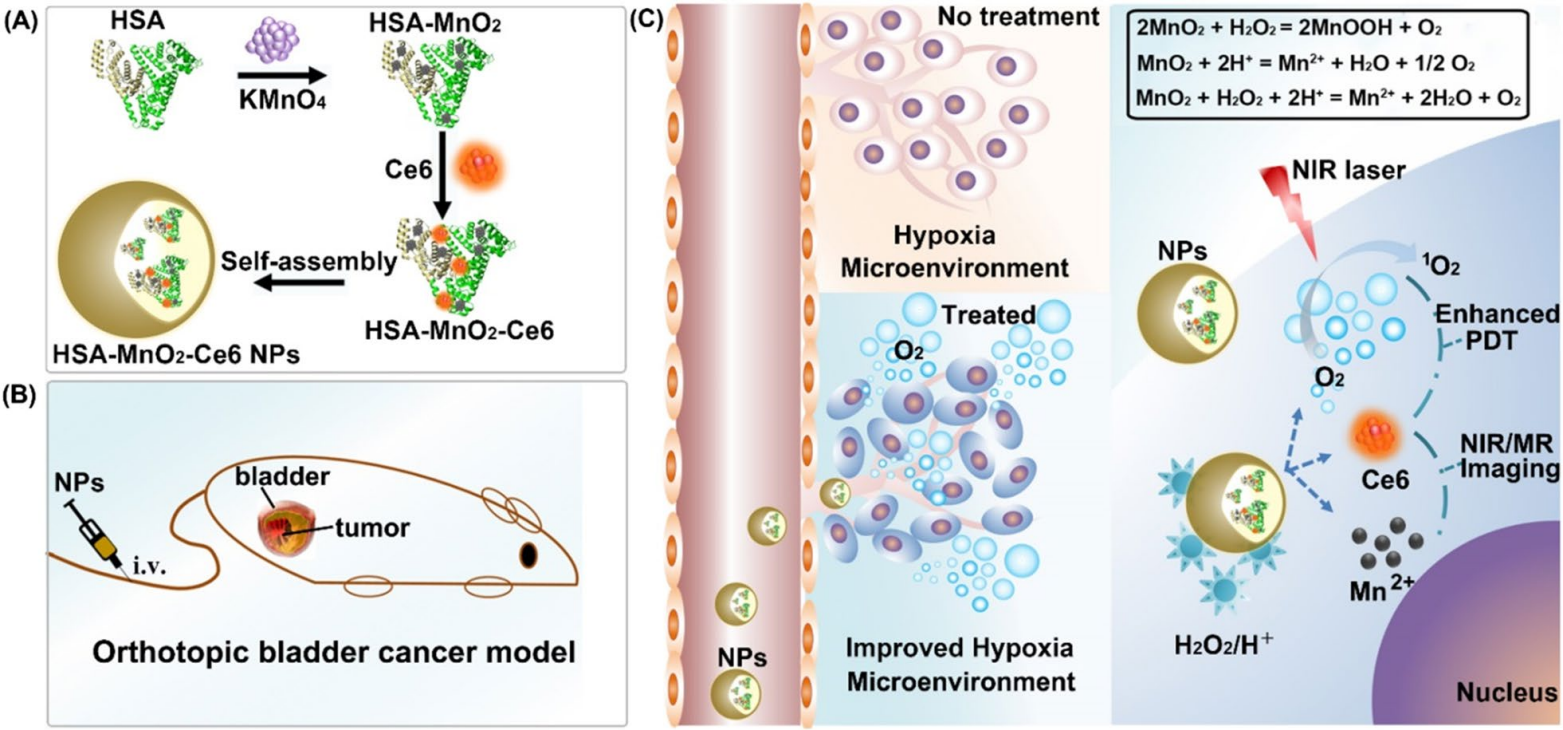
Schematic diagram of HSA-MnO2-Ce6 NPs synthesis (**a**) and enhanced PDT therapy efficiency in orthotopic bladder cancer model (**b**) through ameliorating hypoxia (**c**) [102] ( Copyright 2018, reproduced with permission from Ivyspring)

Phototherapy including photothermal and photodynamic therapy (PDT) has been proposed as a potentially novel treatment modality for a variety of cancers such as lung, gastrointestinal and gynecological malignancies [91–93]. The theoretical base of phototherapy is that a photosensitizer, by absorbing energy from the irradiation of light with certain wavelength, transfers oxygen to reactive oxygen species (ROS), which can cause a cascade of biochemical events and finally lead to apoptosis and the necrosis of cancer cells [94–96]. However, the clinical utilities of PDT are restricted because of the limited penetrating tissue depth or toxicity to normal cells of different lights. Nanotechnology loaded with photosensitive drugs for PDT has attracted much attention in cancer treatment. Promising upconversion NPs loaded with different photosensitizers were evaluated for a synergistic effect of PDT. Zinc(II)-phthalocyanine, as a photosensitizer, could be targeted to a tumor site and enhance the toxic effect of PDT. Mesoporous silica is a delivery system for water-insoluble drugs with features of an adequate surface area and porous structure. Therefore, mesoporous silica-coated NaYF4 NPs were synthesized to incorporate zinc(II)-phthalocyanine into the pores of shells and then used as PDT agent. It was noticed that the zinc(II)-phthalocyanine was activated by red fluorescence generated by the near infrared (NIR) to visible upconversion reaction, but not by the direct irradiation of a NIR laser. Under the exposure of a 980 nm NIR light, cell apoptosis and death were observed when incubated with mesoporous silica NPs, which were able to act as useful tools for PDT [97]. Another photosensitizer, 5-aminoevulinic acid, has disadvantages of hydrophilicity and poor penetration into bladder tissue. To overcome these problems, copolymers were utilized to formulate photosensitizer loaded NPs. The nonamodified 5-aminoevulinic acid significantly improved the photodynamic effect on BC cells [98]. Black titanium dioxide NPs (b-TP-700) were synthesized though the facile calcination method and were demonstrated to improve the tissue absorption of visible light and near-infrared light with a narrow band gap. The NPs exhibit efficient anticancer functions with low toxicity and favorable biocompatibility [99]. In addition, AuNPs were used to attach fibroblast growth factor 1 (FGF1), a recombinant ligand of FGFR, to form nanoconjugates which could be specifically internalized. Upon exposed to near-infrared light, tumor cells overexpressing FGFRs showed significant viability decrease while normal cells were not affected. [100]. Nuernisha et al. encapsulated aggregation-induced emission luminogen (AIEgen, BPN-BBTD) with organic NPs for BC diagnosis and treatment. The system showed high quantum yield (QY) in the second near-infrared (NIR-II) window and great photothermal conversion capability. BPN-BBTD NPs were successfully utilized for fluorescence imaging and photothermal therapy in both orthotopic and subcutaneous bladder tumor models. In addition, the photostability and enhanced retention effect made long-term (32 days) tracing possible (Fig. 8) [101]. Tingsheng et al. prepared O_2_-generating HSA-MnO_2_-Ce6 NPs (HSA, human serum albumin; Ce6, chlorin e6) to ameliorate tumor hypoxia and thus improve the photodynamic effect for bladder cancer treatment. After injection, HSA-MnO_2_-Ce6 NPs induced 3.5-fold O_2_ content change in orthotopic bladder cancer and significantly improved the therapeutic efficacy (Fig. 9) [102].

### Delivery of biological agents

Nanotechnology was also applied in delivering biological agents and targeted drugs. For gene delivery, cationic liposomes were reported to be attractive candidates with features of immunologic tolerance and biocompatibility [103]. The nanotechnology platform was constructed by using cationic liposomes to load the antitransferrin receptor single-chain antibody fragment and RB94, which is a tumor suppressor gene. This nanocomplex presented an effective delivery for RB94 to the tumor site and showed the ability to deliver RB94 and conventional chemotherapeutic drugs for a better inhibition of bladder cancer growth [104]. RNA interference (RNAi) is one of many evolutionary gene therapies as an oligonucleotide-based method. A small interfering RNA (siRNA) can generate gene regulation by degrading messenger RNA to silence the expression, thereby possibly reducing the production of toxic protein. Poly (lactic-co-glycolic acid) (PLGA) NPs modified with chitosan were designed to encapsulate surviving siRNA, aiming to decrease the surviving protein expression and thereby inhibit tumor growth, which were realized by in vivo and in vitro studies [105]. RNA activation (RNAa) is a recent emerging method to selectively overexpress a protein, representing as a new strategy for gene therapy. RNAa refers to small activating RNAs (saRNAs), which are also double-stranded small RNAs like siRNAs. They harbor opposite functions as a result for different targeting. With the advantage of delivering siRNAs technique, dsP21-322 was successfully loaded into lipid nanoparticles (LNP) for intravesical delivery (LNP-dsP21-322–2’F). The system can effectively induce the p21 production and cause cell-cycle arrest and apoptosis both in vivo and in vitro [106]. In addition, NPs could improve the accumulation of chemotherapeutic drugs in tumor tissue and reduce side effects to normal cells. Despite the prospective of targeting to tumor cells, the off-target distribution of NPs in normal stromal cells was still observed and reported to cause damage to tumor-associated fibroblasts (TAF), which then secreted several recombinant proteins and led to stromal reconstruction and tumor cell resistance. Therefore, TAF might partly explain the resistance of NPs delivered drugs in the treatment of desmoplastic BC [107]. To solve this problem, the key action is to inhibit the expression of toxic proteins secreted by TAF. Miao et al. utilized lipid-coated protamine DNA complexes to load plasmids encoding TNF-related factors sTRAIL. The complexes prevented the activation of TAF, and remodel the tumor microenvironment for better efficiencies of repeated nanotherapy [108].These results were encouraging with a significant inhibition of tumor growth and prolonged survival, which indicated that this novel delivery system with gene therapy might be a promising way to cure BC.

## Multifunctional nanoparticles

Besides the aforementioned nanoparticles designed for a specific function in BC management, some researchers also designed nanoparticles with multiple functions which can facilitate the diagnosis and treatment of BC at the same time.

Kepeng et al. loaded folate-modified vincristine into polydopamine-coated Fe3O4 superparticles to form Fe3O4@PDA-VCR-FA SPs. These nanoparticles were applied to bladder cancer theranosis. The superparamagnetism of Fe3O4 endows the system with magnetic resonance imaging capability and vincristine endows it with chemotherapy ability. The system also showed great effect of photothermal therapy when triggered by NIR laser [109]. Suehyun et al. successfully synthesized multifunctional nanoclusters of upconversion nanoparticle (UCNP) and gold nanorod (AuNR) through a PEGylation process. UCNPs endow the system with fluorescent ability under NIR excitation, thus facilitating with tumor detection. They further used EGFR antibody to target bladder cancer cells. Through optoporation-assisted chemotherapy, the system could significantly reduce the effective dosage of cisplatin. The constructed nanoparticles showed great potential in clinical application [110].

## Conclusion

BC is a difficult disease to manage. NMIBC has a high recurrence rate and progression to MIBC is also not rare in clinic, which requires intensive surveillance and aggressive treatment methods. However, the current theranostic methods for BC are unsatisfactory. New diagnostic methods with high specificity and accuracy, and more efficient agents are urgently needed in clinic.

Nanotechnology has great potential in cancer diagnosis and therapy. It enable us create devices with desired chemical, physical and/or biological properties to manipulate cells and signal pathways efficiently, thus assist with the diagnosis and treatment for all kinds of diseases. The management for bladder cancer patients remained almost unchanged for decades with unpleasant curative effect, so exciting improvement may be soon available with nanotechnology. Tremendous success has been made for bladder cancer diagnosis and treatment as mentioned in our review.

All these progression of nanotechnology in the field of bladder cancer may finally contribute to its management and yield the most satisfactory effect together. So we believe all of them are promising and worth further exploration. Besides the application of nanotechnology in BC diagnosis and treatment, it can also help us with the mechanism exploration. Some obstacles for clinical translation and future development directions should also be addressed.

### Nanotechnology and mechanism exploration

Nanotechnologic materials refer to those with nanometer scale size range (1-100 nm) in at least one dimension. Materials at this scale have lots of specific properties from traditional macromolecules [111]. With this size, they can easily interact with elementary biological units like proteins, DNA and RNA [112]. They can also be decorated with cancer specific moieties, protective polymers or imaging agents to endow nanoparticles with specific properties. We may take these advantages of nanoparticles to reveal the underlying mechanisms of tumorigenesis and progression. Azevedo et al. developed glycan affinity magnetic nanoplatforms to enrich glycoproteins in BC patients, thus facilitating the identification of glycobiomarkers in BC patients [113]. Yasui et al. utilized nanowires anchored into a microfluidic substrate for efficient collection of urine extracellular vesicles (EVs), and microRNAs (miRNAs) within the EVs were subsequently analyzed. Through this methodology, they identified urinary miRNAs that could potentially serve as bladder cancer markers [114]. Besides the conventional biological or physiological methods, nanotechnology may serve as a novel platform for mechanism exploration.

### Nanotechnology and clinical translation for BC

Despite the great successful in experiment, the clinical translation of nanotechnology is rather limited. There are some main challenges for this. Biodistribution modulation is one of the main challenges. Immune cells may rapidly clear the NPs entered into the body and hinder the clinical usage. Appropriate modification or strategy is necessary for the prolonged circulation time to accumulate in the target site. Safety concern is also one main challenge. All NPs must be carefully evaluated before clinical trials since these multifunctional and composition complicated nanoparticles may have unexpected effects in human body. Studies in animals cannot translate to clinical studies in human since the heterogeneity between human diseases and animal models. Besides, there are technology challenges like large scale synthesis and performance optimization which are essential for clinical success [115]. NP albumin-bound paclitaxel (nab-paclitaxel) is in clinical trials for both intravesical instillation and intravenous administration. This nanotechnology formulated PTX showed limited side effects and promising therapeutic ability. More clinical trials are ongoing for the application of nanotechnology in BC management (NCT02240238, NCT02009332).

### Future development direction and challenges of nanotechnology-based BC theranosis

To summary, we think successful translation for nanoparticles from bench to bedside requires efforts in the following aspects. First, design nano-drugs with higher efficiency and fewer side effects. This is the foundation for clinical translation since efficacy and safety are two important indicators in clinic. With the development of nanotechnology, more effective nano-drugs are continually being explored and new methods for targeting delivery of NPs are becoming ripe and practical. Second, explore and illuminate the functional mechanisms of nano-drugs in cancer theranostic process. Thorough understanding about the targeted pathways and underlying mechanisms about nano-drugs will greatly facilitate the future design of useful nanoparticles. With clear understanding about the interactions between nanoparticles and biosystems, it will be easier to improve the efficacy and avoid side effects. Third, combine fundamental researches with clinical studies. Clinical trials are the bridges between basic researches and clinical practice, and they are inevitable for clinical translation. More clinical studies are needed for the developed nanosystems come from fundamental studies. Fundamental studies can also help to solve the problems discovered from clinical trials. Close combination of fundamental researches and clinical studies can greatly accelerate the translation process.

Based on these promising results of nanotechnology on diagnosis and therapy of BC, more researches are required in this field to bring solid benefits for BC patients. We have full confidence that nanotechnology combined with comprehensive understanding of pathological mechanisms can usher us into a new age for BC management.

## Data Availability

Not applicable.

## Abbreviations

5-ALA: 5-Aminolaevulinic acid
AuNPs: Gold nanoparticles
BC: Bladder cancer
BCG: Bacillus Calmette-Guerin
BCG-CWS: BCG cell wall skeleton
BLC: Blue light cystoscopy
CIS: Carcinoma in situ
CS: Chitosan
CT: Computed tomography
CTAB: Cetyl trimethyl ammonium bromide
dHPGs: Derivatized hyperbranched polyglycerols
DLS: Dynamic light scattering
Dox: Doxorubicin
DTX: Doxetaxel
EVs: Extracellular vesicles
FGF1: Fibroblast growth factor 1
HA: Hyaluronic acid
HAL: Hexaminolevulinate
HAS: Human serum albumin
HURP: Hepatoma upregulated RNA
LNP: Lipid nanoparticles
MIBC: Muscle invasive bladder cancer
miRNAs: MicroRNAs
MMC: Mitomycin C
MRI: Magnetic resonance imaging
MSNs: Mesoporous silica nanoparticles
NBI: Narrow band imaging
NCs: Nanocubes
NMIBC: Non-muscle invasive bladder cancer
PDT: Photodynamic therapy
PECA: Poly(ethyl-2-cyanoacrylate)
PGON: Poly(guanidinium oxanorbornene)
PLGA: Poly(lactic-co-glycolic acid)
pMCNP: Peptides modified glycol chitosan nanoparticles
PTX: Paclitaxel
QY: Quantum yield
RNAa: RNA activation
RNAi: RNA interference
ROS: Reactive oxygen species
saRNAs: Small activating RNAs
SERS: Surface-enhanced Raman scattering
siRNA: Small interfering RNA
SPIONs: Superparamagnetic iron oxide nanoparticles
SPR: Surface Plasmon resonance
TAF: Tumor-associated fibroblasts
TEP: Telomerase extension product
TS: Telomerase substrate
TUR: Transurethral resection
TURBT: Transurethral resection of Bladder tumor
USPIO: Ultra-small superparamagnetic iron oxide
WLC: White light cystoscopy
